# Gentrification and crime in Buffalo, New York

**DOI:** 10.1371/journal.pone.0302832

**Published:** 2024-06-20

**Authors:** Zhe Zhang, Ashley Barr

**Affiliations:** Department of Sociology, University at Buffalo, SUNY, Buffalo, New York, United States of America; Feroze Gandhi Degree College, INDIA

## Abstract

Since the 1990s, gentrification has significantly changed American urban landscapes. Its implications for crime are under recent scrutiny, particularly in large cities like New York City, Los Angeles, and Chicago. We extend this literature by focusing on the gentrification-crime link in the midsize city of Buffalo, New York using nine years of data from the American Community Survey and the Buffalo Police Department. Examining changes both within tracts over time and changes between gentrified and never-gentrified tracts, we find that gentrification is associated with reduced property crime and is not associated with changes in violent crime. More specifically, in comparing crime trends across tracts, we find that gentrified tracts show a trajectory of declining property crime that mirrors more advantaged tracts, while vulnerable-but-never-gentrified tracts show a U-shaped trajectory of property crime. Looking at within-tract changes, we find that years following gentrification of a given tract have lower property crime rates than years preceding gentrification, independent of the general reduction in crime over time. We discuss the implications of these findings for understanding the intersections between urban processes and crime.

## Introduction

Gentrification has been occurring in the United States since the financial and economic decline in the 1970s. It has been identified as a process that takes place after a period of disinvestment and economic decline and involves in-migration of a new, middle-class population, financial (re)investment in the inner-city neighborhoods, and often racial reconfiguration [1–5], but see Brown-Saracino [6] and Taylor [7] for contrary evidence on racial reconfiguration. Diverse factors drive this process, including government renewal policies, cultural changes, private investments, and immigration pioneers [8–11].

Research has demonstrated that gentrification significantly changes—for better or worse—neighborhood characteristics. It has been associated with growing housing values, increased economic integration [2,12], residential displacements [13], racial composition variations [14], social class transformation [11], and crime rate changes [15]. This latter effect, that of gentrification on crime, however, is still an open question. Some scholars have argued, for instance, that there is a positive relationship between gentrification and crime, highlighting the role of incomplete neighborhood transformations, disruptions of the established social order, and the aggregation of suitable and lucrative targets in gentrifying neighborhoods in increasing crime rates [16,17]. On the contrary, others have argued that gentrification is accompanied by decreasing neighborhood crime rates, potentially through establishing relatively stable areas with an influx of economically advantaged residents [15,18,19]. Macro-level trends support this latter perspective, as the city redevelopment process coincided with U.S. crime changes in the late 20^th^ century—crime rates in major U.S. cities increased from the 1960s to the 1980s and then experienced a considerable drop during the 1990s [20].

These contradictory perspectives on how gentrification affects crime may be attributable to methodological variations in the field. Scholars have distinctive operationalizations of gentrification, use different time intervals to capture gentrification changes (mostly ranging from 5–30 years), and various statistical analysis strategies that vary in the specific questions they are able to answer [5,15,19,21,22]. Further, much of what we know about gentrification and crime, and gentrification more generally, is based on studies of large cities like New York City, Los Angeles, and Chicago. As Ocejo, Kosta, and Mann [23] argue, however, “lessons learned in large cities do not always apply” to smaller cities, and “the urban experience in smaller cities holds the potential to expand our theoretical toolbox for understanding gentrification” (p. 10). Also, these larger cities under scrutiny typically began gentrification processes earlier or amidst a very different crime backdrop than those that can be found in the past decade [15,21,24,25].

The current study builds on past work to examine the association between gentrification and crime at the census tract level across nine years in Buffalo, New York, a mid-sized city whose gentrification story began relatively recently [26,27]. We pay particular attention to the within- and between-tract effects of gentrification on crime. This allows us to address lingering concerns in the literature about the extent to which changes in crime are attributable to changing processes *within* gentrified neighborhoods or are due to differing characteristics *between* gentrified and non-gentrified tracts. We ask four questions in this study. First, how are gentrified tracts different from not-vulnerable (i.e., advantaged) tracts and from vulnerable-but-never-gentrified tracts in their initial crime rates? Second, how does the trajectory of crime differ by gentrification status? Third, within a given tract, how are changes in gentrification status associated with changes in crime? Fourth, among gentrified tracts, how are crime trajectories associated with the timing of gentrification? Although we investigate many questions, each addresses a specific piece of the gentrification-crime puzzle. Consistency across these pieces enables a much clearer picture of the link between gentrification and crime in Buffalo.

### Theoretical frameworks of gentrification and crime

Research to date draws upon social disorganization and routine activities theories to explain the relationship between gentrification and neighborhood crime, but both theories have been used to produce internally divergent hypotheses. That is, mechanisms drawn from both theories have been used to explain why gentrification may either increase or decrease crime. Table 1 summarizes these theoretical mechanisms, which we further explicate below.

**Table 1:** Theoretical mechanisms linking gentrification to crime.

Theory	Crime-promoting Mechanisms	Crime-reducing Mechanisms
Social Disorganization Theory	Increased residential instability Increased ethnic heterogeneity	Decreased concentrated disadvantage
Routine Activities Theory	Increased suitable targets Reduced presence of capable guardians (due to disruption of network)	Reduced presence of motivated offenders Increased presence of capable guardians (due to increase in resources)

As shown in Table 1, one can deduce both crime-promoting and crime-reducing mechanisms from social disorganization theory. Beginning with the former, social disorganization theory contends that the elevated levels of disorder, indicated by concentrated disadvantage, residential instability, and ethnic heterogeneity, can exacerbate neighborhood crime rates [28]. Through this lens, gentrification is a process often accompanied by the intensification of residential instability and ethnic heterogeneity, leading to population displacement, racial transformations, and social network disruptions, further stimulating crime. The flood of new homeowners into gentrifying neighborhoods replaces the original homeowners and renters, who can no longer afford the growing property tax or rent associated with upgraded property values. This residential instability may lead to the disruption of established social networks and social control, further interrupting neighborhood development [29–31]. Further, different socioeconomic statuses between the newcomers and incumbent residents generate social distance between them, impairing a community’s ability to form new social ties and informal social controls [22,32,33]. New residents may have clashes with long-term residents due to dissimilar understandings of normative neighborhood behaviors [34]. Furthermore, since gentrification is often associated with racial transformation [2], as Blau [32] declares, social distrust and distance are more pronounced in gentrifying neighborhoods that involve incoming different racial or ethnic groups. Therefore, a sense of fear and hostility can prevent residents from building collective efficacy, which is a widely acknowledged mediator between neighborhood structural changes and crimes [35,36].

Importantly, as shown in Table 1, inferences drawn from social disorganization theory may also reach the opposite conclusion: gentrification is beneficial to reducing neighborhood crime. With wealthy residents moving into deprived neighborhoods during gentrification, the level of concentrated disadvantage is expected to fall significantly. Through this mechanism, one would expect local crime rates to decrease, as affluent homeowners are disposed to invest greater material and social capital in their living neighborhoods [25]. Also, the improvements to the neighborhood environment and the gain of social resources are beneficial to building neighborhood collective efficacy, which is favorable to reducing crime [37]. Finally, the inflow of homeowners in gentrifying areas contributes to greater residential stability [22].

As with social disorganization theory, arguments derived from routine activities theory [38] have also been used to predict both decreases and increases in crime following gentrification. These countervailing mechanisms are also summarized in Table 1. According to routine activities theory, crimes are likely to reach a higher level when motivated offenders identify suitable targets, with the absence of capable guardians, at the same time and space. Using this theoretical framework, the sudden increase of middle-class residents may serve as suitable targets who possess higher-value goods and property, thereby attracting offenders to their neighborhoods [22]. Further, the long-term residents may be unwilling to act as capable guardians for the middle-class in-movers. The new residents are less protected by social networks from potential crimes, which may further increase their victimization risk.

Conversely, one may use routine activities theory to predict a decrease in crime associated with gentrification. During gentrification, the disadvantaged long-term residents, who are more inclined to become the motivated offender, are displaced, which can reduce crimes in these neighborhoods [22]. Moreover, some studies find that capable guardianship is intensified with the development of gentrification. For example, middle-class in-movers may demand more frequent policing in gentrifying communities [39,40] and have the economic resources to enhance surveillance strategies, such as installing cameras or having security guards [24]. In addition, with more small businesses opening in gentrifying neighborhoods, business vendors serve as “eyes on the street” to monitor outside activities [41].

### Empirical works on gentrification and crime

Consistent with the conflicting predictions made by these theoretical frameworks, empirical analyses also produce mixed findings on gentrification’s impacts on various kinds of neighborhood crimes. A great number of studies have found gentrification is associated with lower crime rates—including homicide, rape, robbery, and aggravated assault [15,19,25,25,42]. Other scholars have identified increased crime rates associated with gentrification. Covington and Taylor [16] asserted that there was a positive association between gentrification and robbery and larceny in Baltimore during the 1970s, as areas with a rapid increase in home values had greater chances of robbery and larceny compared with slowly appreciating neighborhoods. Similarly, research in Los Angeles during the 1990s found that gentrification was related to higher assault and robbery rates, but had no significant association with homicide or rape [17]. Smith [43] also assessed the relationship between gang-specific homicides and gentrification. He found that private-investment-driven gentrification, indicated by the proliferation of coffee shops, was related to decreased gang homicides, while state-based gentrification, measured by the demolition of public housing, was associated with increased gang homicide.

At the same time, some researchers have argued for the short-term positive and long-term negative impacts of gentrification on crime [22,24]. These scholars contend that different periods before and after gentrification have distinctive impacts on crime changes (i.e., a nonlinear relationship). When the process initially starts with spotty occurrences, the disruption effects may be more prominent. Multiple studies linking gentrification with increased risk of crimes [16,17] focus on the period around the late 20^th^ century when gentrification was a sporadic and incomplete process. However, with its continuing expansion in neighborhoods, gentrification was driven by large private corporate investments [24] or state-based public housing programs [44], and this “consolidated” revitalization may undermine the criminogenic condition of neighborhood changes when the population changes in favor of the new gentry [24]. The study period examined here is in the 2010s, thus we expect the disruptive effect of gentrification may be less obvious in Buffalo, implying an even greater reduction in crime under the impacts of “consolidated” gentrification.

Importantly, these studies point to the utility of examining violent and property crime separately, as some mechanisms linking gentrification to crime may be more relevant to one type of crime versus the other. Kawachi et al. [45] show, for instance, that relative deprivation (income inequality) and indicators of low social capital were consistently associated with violent crimes, while only a certain category of property crime—burglary—was associated with both deprivation and low social capital. Similarly, Barnett and Mencken [46] found that resource disadvantage was not related to property crime in nonmetropolitan counties but identified a positive effect of resource disadvantage on violent crimes.

### Current study: Gentrification and crime in Buffalo, New York

Although this study cannot reconcile all the discrepancies in past work, we expand upon this work in two important ways. First, we incorporate a mid-sized American city into the national U.S. gentrification narrative. Gentrification research to date has mostly focused on large-sized, global, and economically successful cities [47]. Studying the causes, patterns, and impacts of gentrification in small cities like Buffalo not only reflects the reality of city redevelopment in smaller city contexts, but also offers the potential to enrich our theoretical framework for comprehending gentrification [23]. Buffalo is a typical American Rustbelt city. Rustbelt cities, like Cleveland, Ohio, and Detroit, Michigan, saw an economic boom in the 1940s. As heightened competition from foreign markets and the lack of investment in the modernization of technology remained, they experienced major deindustrialization and population loss in the latter half of the 20th century [48,49]. Buffalo, in particular, was known for grain and steel since the early 19^th^ century, while deindustrialization resulted in the closure of a huge number of industries and a rise in the unemployment rate in the 1980s. Simultaneously, Buffalo experienced a considerable reduction in population—its metropolitan area lost roughly 58,000 people in the 1980s [50]. Nevertheless, scholars have shown that economic collapse may be a prerequisite for many city neighborhoods to become vulnerable to gentrification [51].

Around 2010, Buffalo witnessed a surge in development initiatives, including the Canalside project, a waterfront development endeavor exceeding $300 million in investment, the expansion of the Buffalo Niagara Medical Campus, and the “Buffalo Billion,” a state-funded initiative to revitalize economic development in the city and broader metropolitan area [52,53]. Moreover, as an indicator of recovery, Buffalo has become an attractive destination for refugees and migrants since 2000—its metropolitan area gained around 21,000 international migrants while losing 22,000 native-born residents [54]. This refugee and immigrant resettlement substantially contributes to Buffalo’s economic reinvigoration by opening up small businesses and keeping the housing market alive [52].

By studying neighborhoods in the mid-sized American city of Buffalo, this study examines gentrification in a place that has continued to go through economic hardships while simultaneously experiencing the traditional hallmarks of gentrification. Buffalo’s relatively late gentrification experiences resonate with numerous old industrial cities (e.g., St. Louis, Missouri [55]) that actively pursue revitalization strategies, making Buffalo a noteworthy exemplar of such urban centers. Further, it examines the gentrification-crime link in a city that began gentrifying relatively recently and amidst a different national crime backdrop than that of larger cities for which gentrification began earlier [15].

Second, we use nine years of data from 79 census tracts in Buffalo to parse out the between- and within-tract effects of gentrification on crime. By attending to both levels of effects, we can assess how much of the variation in crime is due to initial differences between tracts (e.g., in concentrated disadvantage) as opposed to differences within tracts over time (e.g., the extent to which a given tract looked different before and after gentrification). This latter effect is important, as it controls for time-invariant, unmeasured differences between tracts that may make gentrified tracts different from vulnerable-but-not-gentrified and not-vulnerable tracts. We pay particular attention to the potential nonlinearity of both between- and within-tract effects of gentrification on crime given the potential for short-term positive and long-term negative impacts of gentrification on crime [22,24]. Using yearly data, a similar approach to Kreager et al.’s [24] work, we provide a tighter timeline than using the conventional 5-year or 10-year interval [15,16,21,22]. Hence, it allows us to capture the process of gentrification as it is occurring rather than as a before and after snapshot, which longer time interval estimates are more likely to provide.

Using data from Buffalo, this study attends to the dynamic nature of gentrification and its association with crime. More specifically, we predict crime rates in 79 census tracts in Buffalo from 2011 to 2019 using a three-stage analysis. First, we consider between-tract differences in initial crime rates and the trajectory of crime from 2011 to 2019. This stage allows us to answer the question of how gentrified tracts differ from their not-vulnerable and vulnerable-but-never-gentrified counterparts in their initial crime rates and their changes in crime over time. Given that disinvestment and disadvantage is a precondition of gentrification, we expect that gentrified tracts will have higher initial crime rates than their not-vulnerable counterparts and vulnerable-but-never-gentrified tracts (Hypothesis 1). Further, we expect a reduction in crime for all tracts over time given a general decline in crime across the study period [56]. Given that there are competing mechanisms (shown in Table 1) and contradictory evidence for whether gentrification increases or decreases crime, we put forth competing hypotheses for this relationship. If the crime-reducing mechanisms discussed above in Table 1 are stronger than the crime-promoting mechanisms, then we expect to see faster declines among gentrified tracts than among vulnerable-but-never-gentrified tracts (Hypothesis 2a). On the contrary, if the crime-promoting mechanisms discussed above and in Table 1 are stronger than the crime-reducing mechanisms, we expect to see slower declines in crime among gentrified neighborhoods (Hypothesis 2b).

Second, we use an approach similar to Barton [21] to examine the within-tract effects of gentrification on crime. This stage allows us to assess how, within a given tract, changes in gentrification status are associated with changes in crime. These fixed effects control for between-tract differences (i.e., those measured and unmeasured factors that may make some tracts more prone to gentrification) and therefore are not as plagued by selection concerns. Again, given the competing mechanisms discussed above and in Table 1, as well as contradictory evidence regarding the association between gentrification and crime, we put forth competing hypotheses. If crime-reducing mechanisms are stronger than crime-promoting mechanisms, gentrification will be negatively associated with crimes *within a given tract*. That is, years in which a tract is gentrified will have lower crime rates than years in which that same tract was not gentrified, controlling for the general reduction in crime over time (Hypothesis 3a). If crime-producing mechanisms are stronger than crime-reducing mechanisms, however, gentrification will be positively associated with crimes *within a given tract*. That is, years in which a tract is gentrified will have higher crime rates than years in which that same tract was not gentrified, controlling for the general crime trend (Hypothesis 3b).

Finally, we employ a novel operationalization of gentrification to assess within-tract changes over time. Rather than comparing gentrified tracts to other tracts or comparing gentrified years within a given tract to not gentrified years, we use a continuous measure of years from gentrification to assess within-tract changes in crime rates in the years leading up to and following gentrification. This stage allows us to assess how time from gentrification, rather than simply before and after gentrification, impacts crime within a given tract and, hence, attends to potential nonlinearity in the gentrification-crime link. Consistent with the above predictions, if the crime-reducing mechanisms are stronger than the crime-promoting mechanisms, we expect to see a decline in crime leading up to gentrification (simply given the general reduction in crime across the study period) followed by a steeper reduction following gentrification (Hypothesis 4a). If the crime-promoting mechanisms, however, are stronger than the crime-reducing mechanisms, we expect to see the same initial pattern leading up to gentrification but then a slower decline (or even an increase) in crime in the years following gentrification (Hypothesis 4b). How long after gentrification we see this change is an open question, as it relates to the potential short and long-term effects of gentrification on crime [24].

## Method

### Data

We combined two datasets for this study to obtain information about the 79 census tracts in Buffalo from 2011 to 2019. First, we used the public access dataset Crime Incidents by *Open Data Buffalo* (data.buffalony.gov), which includes crime data provided by the Buffalo Police Department. This dataset is updated daily and provides crime incidents reported to the police in Buffalo at the census tract level from 2009 to 2024. Second, we used the American Community Survey 5-year Estimates from 2011 to 2019, which provides information about census tracts for gentrification-related variables, county-level information, as well as neighborhood control variables, including measurements of concentrated disadvantage, residential stability, the percentage of the foreign-born population, and the percentage of the youth population. For brevity, we refer to the ACS sample by the end year of its time window (e.g., 2015 for ACS 2011–2015).

### Dependent variable: Crime rate

**Fig 1:**
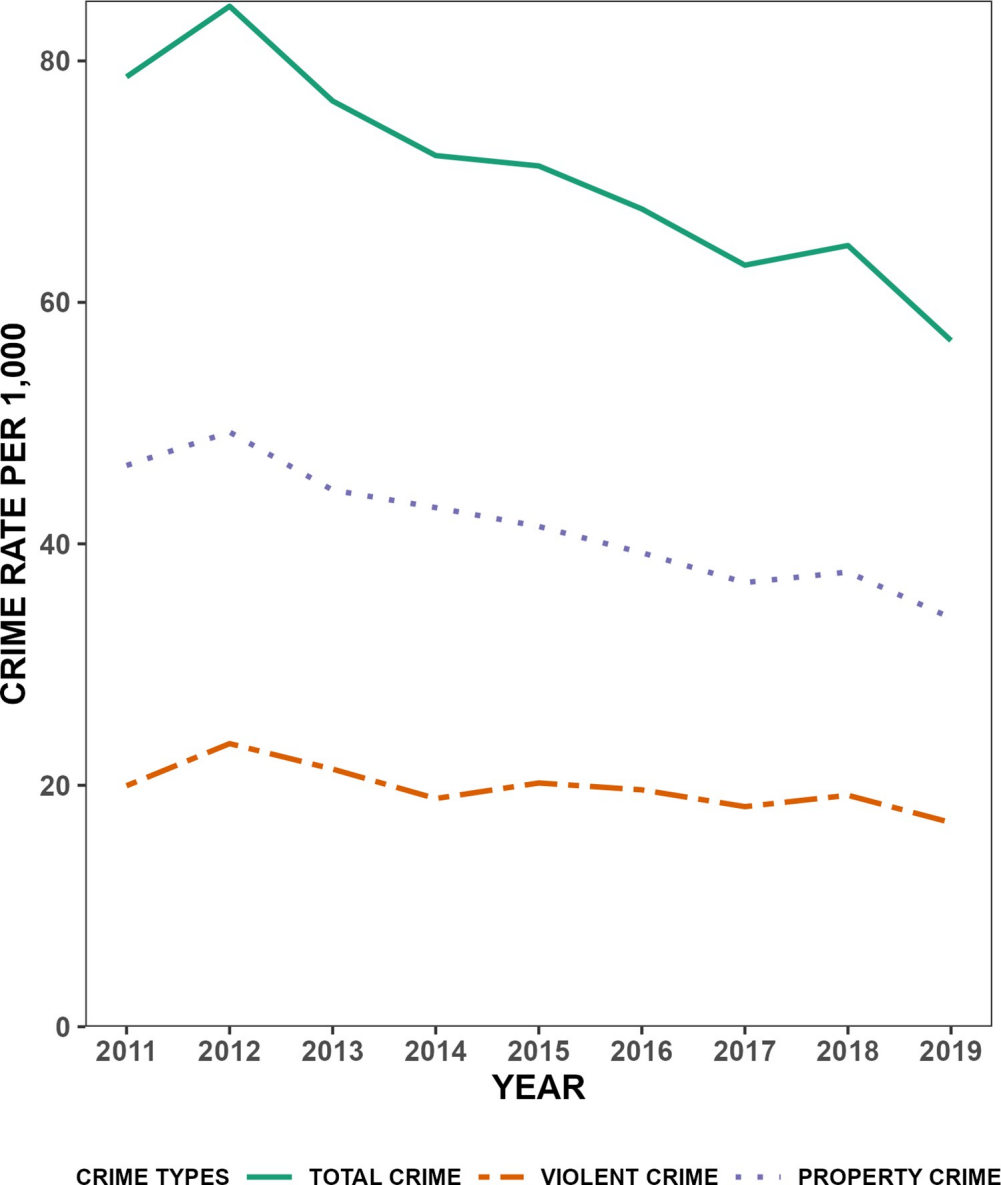
Crime trend in Buffalo from 2011–2019.

Consistent with past work examining gentrification and crime [24], we examine property and violent crimes separately based on standard Uniform Crime Report definitions. The dependent variable is the property crime rate and violent crime rate per 1,000 tract residents in Buffalo per year. Property crimes include breaking and entering, larceny-theft, and motor vehicle theft. Violent crimes include homicide, robbery, and aggregated assault, classified in the same way as Barton [20]. Incident-level information on the total counts of criminal behaviors in Buffalo was aggregated to 2010 Census tract boundaries. Fig 1 shows that the average tract experienced an overall decline in total crime, property crime, and violent crime between 2011 and 2019. For analytic models, we employed a lead variable of crime rate to achieve appropriate time-ordering. Such an approach is mathematically similar to lagging the independent variables. In this case, however, a lead is preferred given that it allows us to retain the 2011 data in the study.

### Independent variable: Gentrification

**Fig 2:**
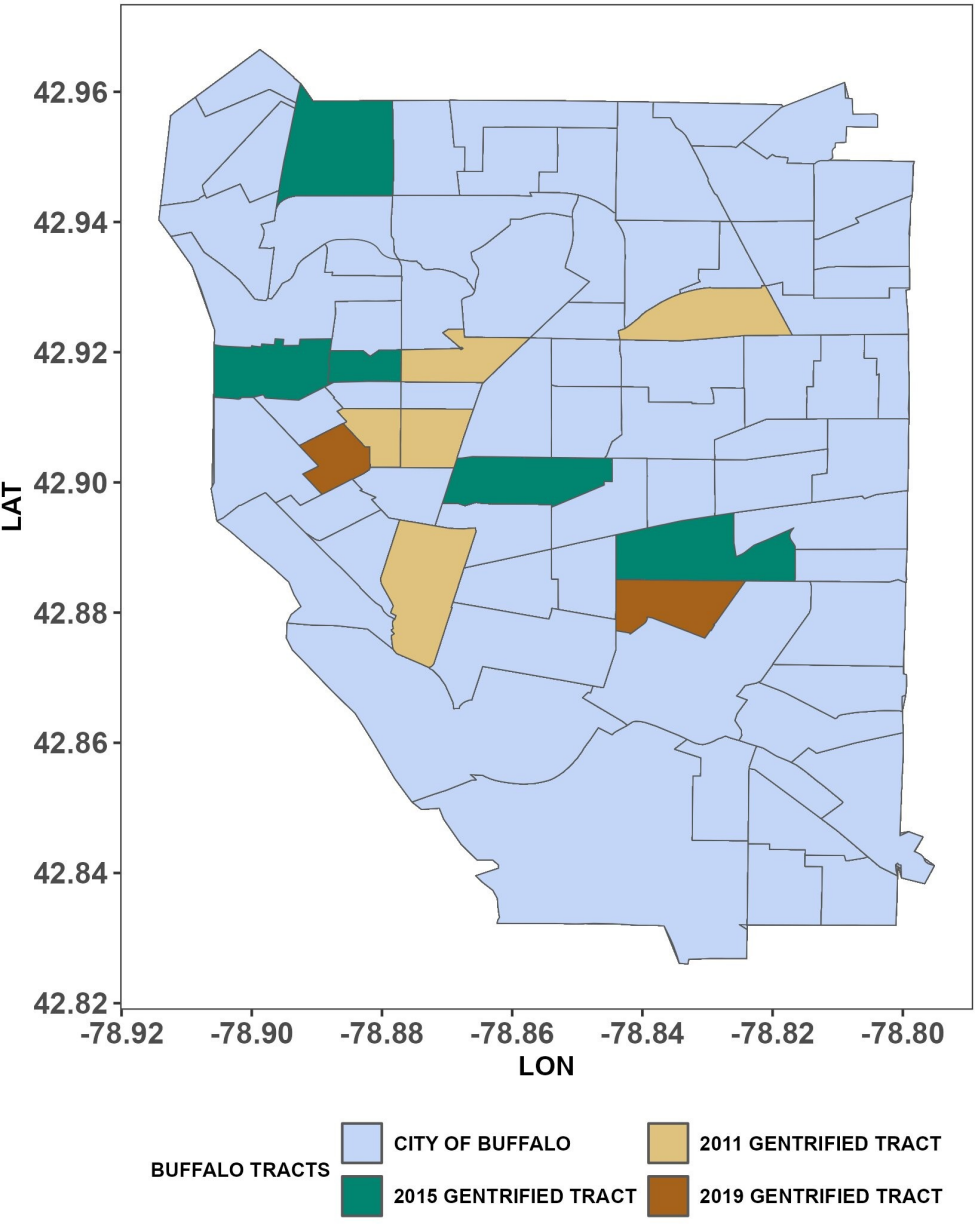
Gentrified tracts in Buffalo in 2011, 2015, and 2019.

Data for the independent variables from 2011 to 2019 were gathered from the 5-year American Community Survey estimates about the 79 tracts defining Buffalo. We follow Barton et al.’s [21] gentrification measurement to identify gentrified tracts in a given year in Buffalo from 2011 to 2019 with slight modifications due to data availability. Census tracts in Buffalo were first identified as vulnerable to gentrification if they meet at least *three out of four* of the following criteria in the previous year of the targeted year (e.g., whether the tract is vulnerable to gentrification in 2018 when the targeted year is 2019): 1. the percentage of residents living in poverty was above the 40th percentile county level; 2. the percentage of residents with a college degree or higher was below the 40th percentile of the county; 3. the percentage of renters was above the county level; 4. the percentage of non-Hispanic White residents was below the county level. Then, these tracts classified as vulnerable to gentrification were identified as gentrified during the targeted year (e.g., 2019) if they experienced all of the following: 1. an increase in the percentage of residents with a college degree greater than the average change at the county level; 2. an increase in median household income greater than the average increase for the county; 3. an increase in the percentage of non-Hispanic Whites greater than the county average; and 4. an increase in median gross rent greater than the county average. Fig 2, created from the R package tidycensus (version 1.3.2; [57]), shows the spatial distribution of tracts that gentrified at the start (2011), the middle (2015), and the end of the study period (2019).

It should be noted that while we operationalize gentrification using county-level comparisons, as is typical in research on gentrification [21], we also operationalized gentrification by using city-level comparisons. These analyses show a similar pattern of results and are included as supporting information. Given the relative disadvantage of Buffalo compared to Erie County, however, city-level comparisons produce fewer gentrified tracts. This is because more tracts (50.7% vs. 10.4%) are considered “not vulnerable” from the start, as they are slightly above the city mean on markers of vulnerability (but still disadvantaged relative to the county). Nonetheless, results are consistent across operationalizations.

From these year-to-year baselines, we created three different measures of gentrification—one between-tract measure and two within-tract measures. First, we created a three-category *time-invariant (e*.*g*., *between-tract) measure of gentrification*. If a tract was ever gentrified from 2011 to 2019, the tract was coded as gentrified. If a tract was vulnerable to gentrification based on the aforementioned criteria but not gentrified across the study period, it was coded as vulnerable-but-not-gentrified. If a tract was neither vulnerable nor gentrified across the study period, it was coded as not-vulnerable. Second, following Barton et al’s [21] approach, we used another binary measure that examines, within a given tract, if the tract is gentrified or not each year during the study period. This is the first *time-varying measure of gentrification* within a tract. In this measure, once a tract is gentrified, it is coded as gentrified for the rest of the periods given that these data assess year-to-year change, and a tract flipping back and forth from gentrified to not on a yearly basis is not theoretically feasible. For example, if a given tract was not gentrified in the years between 2011 and 2019, all tract-years were coded as 0; if it was not gentrified until the year 2014, it was coded as 0 before 2014 and was coded as 1 in 2014 through 2019. Finally, we created a new, continuous *time-varying measure of gentrification* that assessed time from gentrification within a given tract. We coded the first year of gentrification as 0 and the years around it as plus or minus the number of years from gentrification. For example, if a tract was gentrified in 2014, one year (the year of 2013), two years (2012), and three years (2011) before the gentrified year was set to -1, -2, and -3, respectively. Similarly, for the same tract, every one year after the gentrified year, the tract was set to a corresponding positive unit value. Using the same example, one (the year 2015), two (2016), three (2017), four (2018), and five years (2019) after the gentrified year (2014) were set to 1, 2, 3, 4, 5 in the variable marking years from gentrification, respectively. Unlike the first two measures, this measure applied only to gentrified tracts. In the results, we make apparent the different interpretations and utility of these measures.

### Control variables

We used four control variables in all analyses. All were measured as between- and within-tract variables. At the within-tract level, each control variable is time-varying and measured every year during the study period; at the between-tract level, each control variable is averaged for a given tract during the nine-year period. We created indices for *concentrated disadvantag*e and *residential stability* using 2011–2019 ACS 5-year estimates data. These two indices were created to account for endogeneity since gentrification often occurs in socioeconomically disadvantaged neighborhoods. The *concentrated disadvantage* measure was created across all tracts by taking the average of the standardized values for the percentage of residents receiving public assistance, the percentage of unemployment, the percentage of female-headed households, the percentage of people in poverty, and the percentage of blacks. The *residential stability* index includes the average of the standardized values of the percentage of the population who lived in the same home for at least 5 years and the percentage of owner-occupied homes for each tract. We also controlled the percentage of *foreign-born* to account for ethnic heterogeneity, and the percentage of the *youth population* (15–24) was also included to control for the population at the greatest risk of offending or being victimized [21]. We consider all controls at both the tract level and the year level to distinguish between contextual effects and within-tract effects.

We also tested for spatial dependence in our data to assess the need for spatial controls. We followed the same protocol as Kreager et al. [24] to assess spatial error and spatial lag using GeoDa for total crime, property crime, and violent crime. Neither spatial error nor spatial lag was statistically significant for the total crime, property crime, or violent crime. We do not include spatial controls in models of property crime.

### Analytical strategy

To examine the impacts of gentrification on property and violent crime in Buffalo, New York, we employed a series of multilevel, random-intercept-only Poisson regression models. This approach is consistent with other analyses of crime rates, which indicates the number of crimes per 1,000 residents [58]. Coefficients therefore represent the log of the expected count of crimes per 1,000 residents given a 1-unit change in the independent variable. These models parsed between- and within-tract effects of gentrification on crime, independent of the general trend of declining crime rates and other time-varying and time-invariant neighborhood controls. These models allowed us to see how much of the gentrification effect on crime was due to differences between gentrified and other tracts (e.g., the between-tract effect), and how much was due to change over time within a given tract (the within-tract effect).

For both property and violent crime, we ran four different models to answer our research questions. The first model, using the first *time-invariant* gentrification variable, examined the initial crime rate differences between tracts of different gentrification status—whether gentrified tracts have significantly higher initial crime rates than vulnerable-but-never-gentrified tracts or not-vulnerable tracts. This model also examined the general time trend and included a linear and quadratic year variable to assess potential nonlinear crime trends. The second model added an interaction term between the time-invariant gentrification variable and year to examine differences in crime trajectories between gentrified, vulnerable-but-not-gentrified, and not-vulnerable tracts. This model, absent controls for simplification, is illustrated in Eq 1.

$$Ln\;{Crime\;Rate}_{t+1j}=\gamma_{00}+\gamma_{01}{Ever\;Gentrified}_{j}{+\gamma_{02}{Vulnerable}_{j}+\gamma_{10}{Year}_{tj}+\gamma }_{20}{{Year}^{2}}_{tj}{+\gamma_{11}{Ever\;Gentrified}_{j}{Year}_{tj}+\gamma }_{21}Ever\;{Gentrified}_{j}{{Year}^{2}}_{tj}+{\;\gamma_{12}{Vulnerable}_{j}{Year}_{tj}+\gamma }_{22}{Vulnerable}_{j}{{Year}^{2}}_{tj}+\mu_{oj}+r_{tj}$$ (1)

In Eq 1, *Ln Crime Rate*_*t*+1*j*_ represents the outcome—the log of the expected count of crimes per 1,000 residents—for time *t+1* in tract *j*. *γ*_00_ indicates the mean outcome across never-vulnerable tracts at the start point. *γ*_01_ and *γ*_02_ indicate the mean crime difference between ever-gentrified and never-vulnerable tracts and between vulnerable and never-vulnerable tracts, respectively. *γ*_10_ and *γ*_20_ indicate the average linear and quadratic growth rate across never-vulnerable tracts, and *γ*_11_ through *γ*_22_ indicate the difference in growth rates between ever-gentrified and never-vulnerable tracts and between vulnerable and never-vulnerable tracts, respectively. *μ*_*oj*_ offers the deviation of tract *j*’s mean from the grand mean (the random intercept), and *r*_*tj*_ indicates the deviation of time *t* from its tract mean across years.

The third model, using the first *time-varying measure of gentrification*, examined the association between gentrification and crime within a given tract. That is, within the same tract, did years in which the tract was gentrified show higher or lower crime rates, controlling for the between-tract effect and general time trend in gentrification? In this third model, between- and within-tract effects are captured within a single model, as shown in Eq 2.

$$Ln\;{Crime\;Rate}_{t+1j}=\gamma_{00}+\gamma_{01}{Ever\;Gentrified}_{j}{+\gamma_{02}{Vulnerable}_{j}+\gamma_{10}{Year}_{tj}+\gamma }_{20}{{Year}^{2}}_{tj}{+\gamma }_{30}{Gentrified}_{tj}+\mu_{oj}+r_{tj}\;$$ (2)

Compared to Eqs 1 and 2 adds the time-varying (within-tract) gentrification measure. Here, *γ*_01_ and *γ*_02_ still indicate the contextual effects, the mean crime difference between ever-gentrified and never-vulnerable tracts and between vulnerable and never-vulnerable tracts, respectively. Whereas *γ*_30_ indicates the average difference between gentrified and non-gentrified years in tract j.

Finally, the fourth and final model restricted the sample to gentrified tracts and used a unique variation of the gentrification variable. It included the continuous time-varying measure of gentrification that assessed *years from gentrification* within a given tract. The model investigated, among gentrified tracts, whether time from gentrification mattered for crime, independent of year gentrified. As never-gentrified tracts did not have a score on time from gentrification, they were excluded from this final model. This model is shown in Eq 3.

$$Ln\;{Crime\;Rate}_{t+1j}=\gamma_{00}+\gamma_{01}{Year\;gentrified}_{j}+\gamma_{10}Years\;from\;{gentrification}_{tj}+\gamma_{20}{{Years\;from\;gentrification}^{2}}_{tj}+\mu_{oj}+r_{tj}\;$$ (3)

In Eq 3, *Ln Lead Crime Rate*_*t*+1*j*_ again represents the crime outcome for time *t+1* in tract *j*. *γ*_00_ now indicates the mean outcome for gentrified tracts during the year of gentrification. *γ*_01_ indicates the difference in expected crime rate between a tract gentrified in one year versus a tract gentrified a year later (i.e., the linear effect of time). *γ*_10_ and *γ*_20_ indicate the average linear and quadratic growth rate of years from gentrification; that is, independent of which year a tract became gentrified, what is the relative increase or decrease in the expected crime rate for each year beyond a given tract becoming gentrified? *μ*_*oj*_ and *r*_*tj*_ still represent the deviation of the tract *j*’s mean from the mean across tracts and the deviation of time *t* from the tract mean.

These different models are not simply different analytical strategies; they help to answer fundamentally different questions based on different units of analysis. For instance, as indicated in Eq 1, between-tract effects concern differences between gentrified and not-gentrified *tracts*, whereas within-tract effects, as added to Eqs 2 and 3, concern differences between gentrified and not gentrified *years* within a given tract. The approach used here allows us to assess both between- and within-tract effects of gentrification on crime within the same model. It is not the case that these effects always coincide. For instance, gentrified tracts may have lower crime, on average, than their vulnerable peers, but changes in gentrification status within the tract may not matter or may matter differently. When these effects do coincide, however, evidence for causation is stronger.

## Results

Table 2 presents descriptive statistics for gentrification measures and neighborhood control variables. These statistics for gentrification measures indicate that there were 5 tracts (6.49% of all Buffalo tracts) that qualified as gentrified in 2011, and about 18% of all Buffalo tracts were gentrified in 2015. By the end of 2019, about 35% of Buffalo tracts were gentrified. Thus, gentrification has been expanding during this study period in Buffalo.

**Table 2:** Descriptive statistics for neighborhood variables at the tract level from 2011 to 2019.

Gentrified tracts	2011	2012	2013	2014	2015	2016	2017	2018	2019
N	5	7	8	9	14	16	20	25	27
Percent of all tracts	6.49	9.09	10.39	11.69	18.18	20.78	25.97	32.47	35.06
Concentrated disadvantage	-0.67	-0.48	-0.11	-0.08	0.05	-0.15	-0.28	-0.31	-0.32
Residential stability	0.19	0.26	0.04	0.01	-0.03	0.00	0.06	-0.03	0.07
% Foreign-born	2.62	2.34	5.01	4.35	4.19	4.12	4.68	5.09	5.48
% Youth	18.79	16.68	16.84	18.98	15.91	15.97	16.28	17.05	16.55
Vulnerable tracts	2011	2012	2013	2014	2015	2016	2017	2018	2019
N	64	62	59	59	48	48	43	40	38
Percent of all tracts	83.12	80.52	76.62	76.62	62.34	62.34	55.84	51.95	49.35
Concentrated disadvantage	0.22	0.28	0.34	0.27	0.36	0.33	0.37	0.27	0.25
Residential stability	-0.07	-0.10	-0.15	-0.19	-0.17	-0.20	-0.28	-0.22	-0.19
% Foreign-born	2.80	3.28	3.21	3.50	3.91	4.02	3.98	3.31	3.52
% Youth	16.97	17.68	17.34	16.98	17.08	16.76	16.08	14.42	14.35
Never vulnerable tracts	2011	2012	2013	2014	2015	2016	2017	2018	2019
N	8	8	10	9	15	13	14	12	12
Percent of all tracts	10.39	10.39	12.99	11.69	19.48	16.88	18.18	15.58	15.58
Concentrated disadvantage	-1.18	-1.14	-0.99	-1.02	-0.92	-1.09	-1.09	-1.12	-1.16
Residential stability	0.86	0.81	0.76	0.80	0.45	0.60	0.48	0.41	0.51
% Foreign-born	1.68	1.35	1.23	1.39	1.59	1.49	2.02	2.69	1.76
% Youth	13.30	12.95	12.93	13.64	13.94	12.74	12.30	12.76	11.80

Also shown in Table 2 are the tract characteristics for ever-gentrified tracts, vulnerable-but-never-gentrified tracts, and never-vulnerable tracts. On average, and as expected, concentrated disadvantage is lowest in never vulnerable tracts, followed by gentrified tracts, and then vulnerable tracts. The reverse patterns are true for residential stability. As young people and foreign-born can be key populations driving gentrification, gentrified tracts are highest on these indicators, followed by vulnerable tracts and never-vulnerable tracts.

Before conducting regression analyses, we examined the intra-class correlation (ICC) to calculate how much of the overall variation in crime rates lies between tracts and within tracts. The ICC indicated that about 86 percent and 84 percent of the unconditional variation in property and violent crime was between tracts. Hence, most of the variation in crime across the study period was between tracts rather than within tracts over time.

Importantly, none of our gentrification measures were significantly associated with violent crime, so we do not show models predicting violent crime (yet these are available from the authors upon request). All findings presented are consistent with those using a total crime (property plus violent crime) measure, as well, likely driven by effects on property crime. Hence, we show and discuss only models for property crime. Table 3, then, analyzed the between-tract and one of the within-tract effects of gentrification on property crime rates among all Buffalo tracts from 2011 to 2019. Model 1 in Table 3 regressed property crime rates on gentrification status among all Buffalo census tracts to test whether gentrified tracts have higher or lower initial crime rates than vulnerable-but-never-gentrified tracts or not-vulnerable tracts. As shown in Model 1, on average and controlling for general neighborhood characteristics, gentrified tracts have higher initial property crime rates compared with their vulnerable-but-never-gentrified counterparts (β = 0.265, p<0.01). Although not shown in the model, supplemental analyses also showed that gentrified tracts have higher initial property crime rates than their not-vulnerable counterparts (β = 0.284, p<0.05). More directly, incident rate ratios (not shown) indicate that gentrified tracts are expected to have a crime rate that is about 30% higher than both their not-vulnerable and their vulnerable-but-not-gentrified peers. This pattern is partially consistent with Hypothesis 1. Also shown in this model is the general property crime trend across the study period, which indicates a nonlinear crime trend with a general decline within the study period.

**Table 3:** Random intercept models predicting property crime rates among all tracts.

	Model 1	Model 2	Model 3
**Tract-level Predictors**			
Gentrification stage (ref = vulnerable, never gentrified)			
Gentrified	0.265**	0.184	0.310**
	(0.094)	(0.104)	(0.095)
Not vulnerable	-0.020	-0.109	-0.010
	(0.095)	(0.121)	(0.095)
Year	-0.097***	-0.128***	-0.095***
	(0.011)	(0.014)	(0.011)
Year^2^	0.007***	0.011***	0.007***
	(0.001)	(0.001)	(0.001)
Gentrified x Year		0.070**	
		(0.022)	
Not vulnerable x Year		0.087	
		(0.050)	
Gentrified x Year^2^		-0.009***	
		(0.002)	
Not vulnerable x Year^2^		-0.011*	
		(0.005)	
**Time-varying predictors**			
Gentrified in a given year			-0.082**
			(0.027)
Constant	3.960***	3.996***	3.949***
	(0.119)	(0.121)	(0.119)

* p<0.

** p<0.01

*** p<0.001. N = 684 tract-years across 76 tracts.

Notes. Standard errors in parentheses. Models control for time invariant and time -variant measures of concentrated disadvantage, residential stability, % foreign born, and % youth.

**Fig 3:**
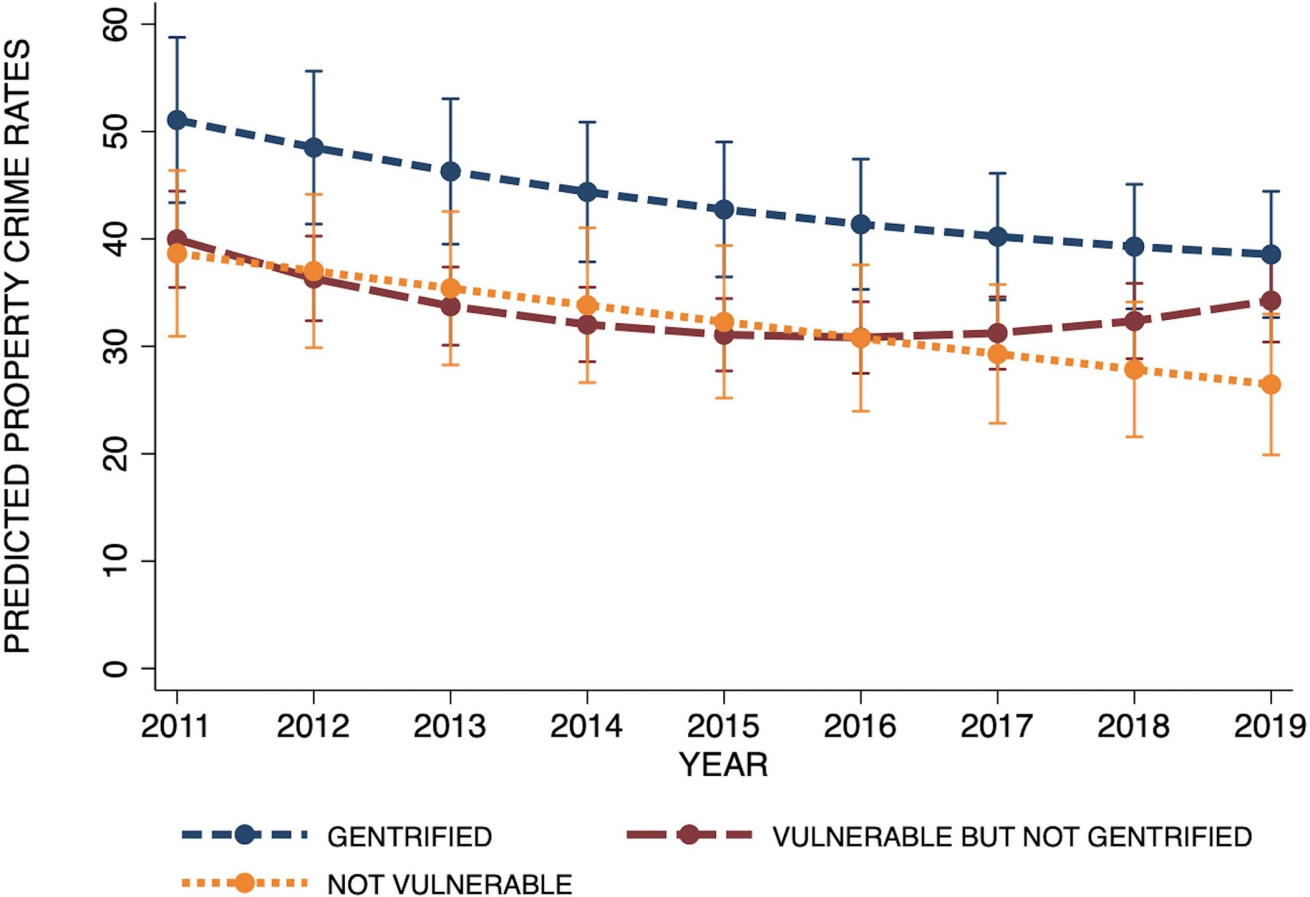
Property crime trajectories among tracts by gentrification status.

Model 2 in Table 3 investigated whether the property crime trajectory across the study period varied by gentrification status by interacting the time-invariant gentrification measure with year and its quadratic counterpart. The significant interaction effects suggest that the crime trend varies by gentrification status. As shown in Fig 3, and consistent with Model 1, gentrified tracts start off with higher property crime rates than their vulnerable-but-never-gentrified and their not-vulnerable counterparts, and they stay higher across the study period. Yet, also evident is that the decline in crime among gentrified tracts mimics the decline among not-vulnerable tracts in that it is stable, whereas the trend for vulnerable-but-not-gentrified tracts is curvilinear, with a decline followed by an increase. This is consistent with Hypothesis 2a and the overall crime-reducing effect of gentrification.

Thus far, the between-tract effects show that trends in crime across the study period differ by gentrification status, but these effects may be subject to selection bias. Gentrified tracts, for instance, may be different from vulnerable-but-not-gentrified tracts in unmeasured ways. Within-tract effects can help rule out some of this bias by using a given tract at a prior time point as its own control. Hence, Model 3 added the time-varying measure of gentrification indicating whether a tract was gentrified in a given year. The coefficient of the time-varying gentrification measure indicates that, within a given tract, years after which the tract was gentrified showed lower property crime rates than years prior to gentrification (β = -0.082, p<0.01), independent of the general time trend, between-tract differences, and time-varying neighborhood characteristics. Again, this result is consistent with Hypothesis 3a and the general crime-reducing effects of gentrification. Also, the coefficient of the within-tract gentrification status has a smaller magnitude compared with the gentrification status between tracts (β = 0.310, p<0.01), as was expected given that most of the variation in crime lay between tracts rather than within tracts over time.

**Fig 4:**
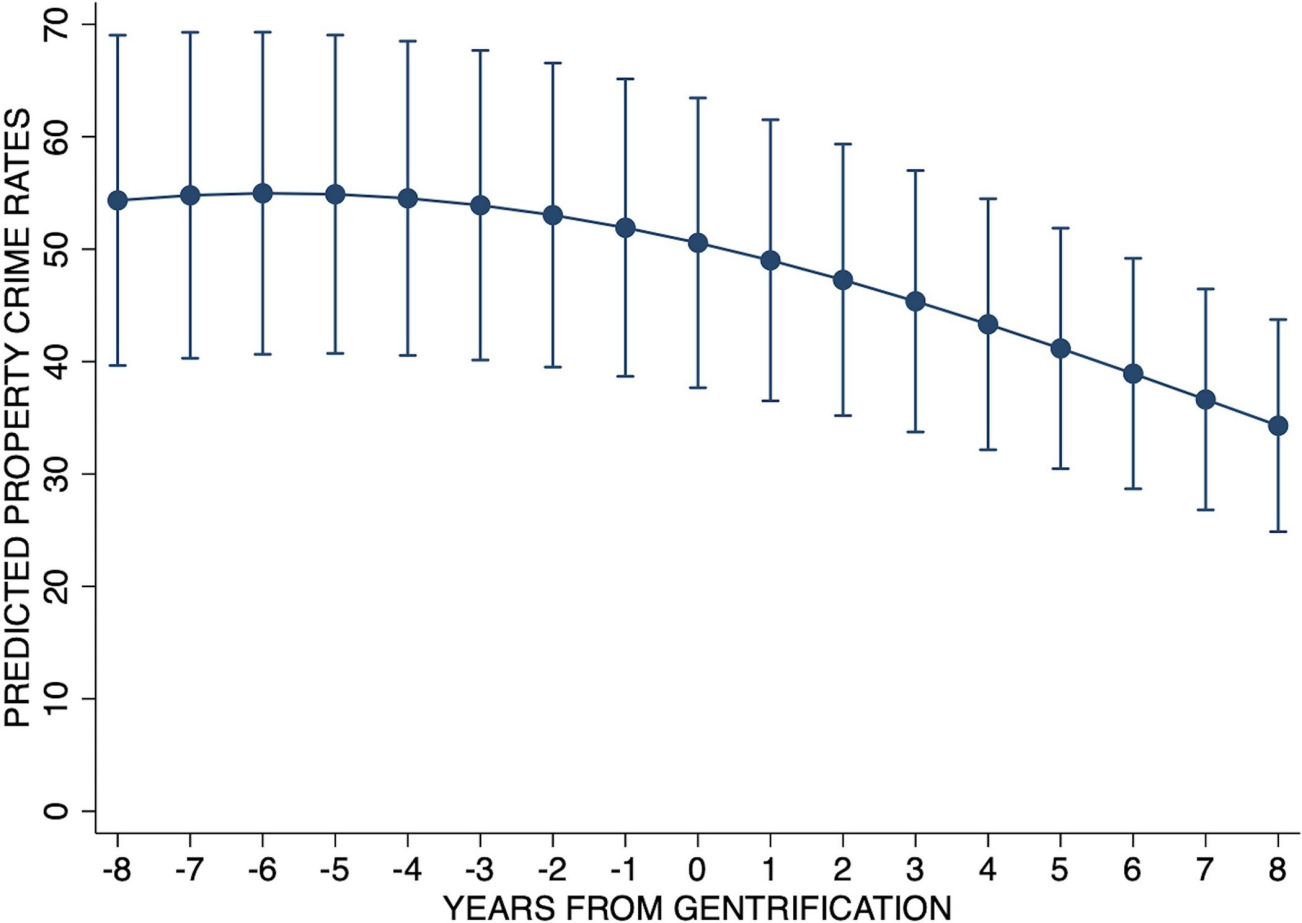
Property crime rate by time from gentrification among gentrified tracts.

Table 4 shows the results from the property crime model employing the continuous, time-varying measure of years from gentrification. Given that only gentrified tracts have a measure of time from gentrification, this model included only gentrified tracts (N = 27). By assessing time from gentrification within a given tract, this model attends to the potentially nonlinear nature of gentrification and addresses the gradual time effect of gentrification that is not fully captured in the year-to-year binary gentrification variable. Both years from gentrification and its quadratic term were examined to assess potential nonlinearity, and, as shown in this model, both terms were statistically significant (β_linear_ = -0.029, p<0.001; β_quadratic_ = -0.002, p<0.001). Specifically, this model indicated an accelerating negative effect of time from gentrification on crime. This acceleration can be seen in Fig 4, where we see a slow decline in property crime among gentrified tracts prior to gentrification followed by a steeper decline as a given tract moves toward and then beyond the point of gentrification. Again, this pattern of results is supportive of the general crime-reducing effect of gentrification indicated in Hypothesis 4a.

**Table 4:** Random intercept model predicting property crime rates among gentrified tracts.

	Property Crime Rate
Years from gentrification	-0.029***
	(0.005)
Years from gentrification^2^	-0.002***
	(0.001)
Year gentrified	-0.065
	(0.044)
Constant	135.553
	(89.101)

N = 241 tract-years across 27 gentrified tracts.

Standard errors in parentheses.

* p<0.05

** p<0.01

*** p<0.001.

Note: Model controls for time-varying measures of concentrated disadvantage, residential stability, % foreign born, and % youth.

## Discussion

This study examined the relationship between gentrification and crime rates in 79 census tracts in Buffalo, New York from 2011 to 2019. Across three different specifications of gentrification, each answering a slightly different question about the link between gentrification and crime, the answer was consistent: although gentrified tracts generally had higher rates of property crime given that disinvestment and disadvantage are precursors to gentrification, gentrification itself was associated with reduced property crime both within and between tracts over time. That is, gentrified tracts saw a more stable decline in property crime rates across the study period than their vulnerable-but-not-gentrified counterparts. This decline mimicked that found in not-vulnerable tracts. Further, within a given tract, gentrification was associated with reduced property crime, and among gentrified tracts, years closer to and following gentrification saw an acceleration of the general decline in property crime independent of where in the study period gentrification occurred.

However, no association between gentrification and violent crime was found in these data. Previous literature shows mixed findings on the relationship between gentrification and different types of violent crime [17,19,43]. For example, Barton et al. [21] found that gentrification was not associated with variation in total or gang homicide, but it was positively associated with non-gang homicide. Also, considering the overall lower level of violent crimes compared to property crimes, the 9-year period in our study may not be long enough to capture modest violent changes. Further, our study centers on a mid-sized city, where the incidence of violent crimes may be too limited for an examination of their association with gentrification. These indicate that future research studying longer periods in mid-sized cities is needed.

The result regarding the higher initial property crime rates in gentrified tracts from our first model set the tone for this study. Initially, property crime was more likely to happen in gentrified tracts than in not-vulnerable tracts or in vulnerable-but-not-gentrified tracts, controlling for neighborhood characteristics. The higher initial property crime rates in gentrified tracts are likely indicative of the decline in these neighborhoods before gentrification. Importantly, however, these differences in initial property crime rates were independent of the neighborhood characteristics measured here and typically thought to explain away these differences (e.g., concentrated disadvantage). Thus, there may be more neighborhood heterogeneity between tracts at different gentrification statuses, and thus more selection mechanisms that have previously been attended to, that should be explored. For instance, vulnerable tracts experiencing high crime may be especially subject to intense policing and more punitive treatments, especially when there is an initial high concentration of minorities, thus enforcing conceptions of public order may spur external initiatives for revitalization [59,60].

Beyond these initial differences between tracts, all other models indicate a crime-reducing effect of gentrification, at least for property crime. The steeper crime-decreasing trend in gentrified tracts compared to their vulnerable-but-not-gentrified counterparts, the negative within-tract gentrification effects, and the accelerating, negative effect of time from gentrification on crime all tell a consistent story of the negative relationship between gentrification and property crime in Buffalo, New York from 2011 to 2019. Moreover, the nonlinear within-tract gentrification-crime relationship suggests that the effect of gentrification on reduced property crime takes time to appear—it can be slower at first while a tract is in the process of gentrifying and more evident with the expansion of gentrification. This is consistent with Kreager et al.’s [24] findings—though they found that, from the 1980s and 2000s, early gentrification was linked to small increases in crime and gentrification in its more established form was negatively related to crime. Considering the contemporary period in Buffalo, gentrification was likely to reach its consolidated form of revitalization, which is the later gentrification stage that Kreager and his colleagues [24] identified.

The consistent findings across three variations of models are particularly compelling given the unique context of gentrification in Buffalo. For instance, compared with other highly gentrified cosmopolitan cities (e.g., New York City, Boston, and San Francisco.), Buffalo has a relatively short history of gentrification. Buffalo’s gentrification process started in the 1990s and has expanded since the 2000s [52]. By 2019, less than 40% of its census tracts were gentrified. Among these gentrified tracts, within the nine-year period, they were gentrified for less than a decade at the longest. And still, we saw a consistent negative association between gentrification and property crime across all models measuring change over time. If it is the case that crime-reducing effects take time to appear since gentrification is a dynamic and gradual process, the effects found here may be relatively conservative estimates. Still, our findings need to be replicated with other operationalizations of gentrification and in other mid-sized cities.

Generally, our findings accord well with studies using contemporary data and support the notion that the crime-reducing mechanisms drawn from social disorganization and routine activities theories may be stronger than the crime-promoting mechanisms, at least for property crime (although we also found no support for a positive relationship between gentrification and violent crime). The negative within-tract association between property crime and gentrification—that years following gentrification within a given tract had lower property crime rates than years prior independent of the time trend—is consistent with Barton [15], MacDonald and Stokes [18], and Papachristos [19] findings. Also, the accelerated decline in crime in a given tract following gentrification is consistent with Kreager et al’s [24] finding of the negative link between crime and the consolidated form of gentrification. However, compared with Lee’s [17] work in Los Angeles, which suggests that gentrification leads to increases in assaults, robberies, automobile thefts, and thefts from automobiles in the short term, our study indicates that the relationship between gentrification and crime can vary depending on specific study sites, focusing on mid-sized cities can broaden the understanding of this relationship.

Still, there are several limitations in this study. First, our study period from 2011 to 2019 was relatively short, and our study cannot examine the tracts that were gentrified before 2011. Those tracts could have been fully gentrified before 2011 but were regarded as not vulnerable because they were never at risk of gentrification during the study period. Yet, due to the limitation of crime data—no accurate data before 2009—we were not able to examine the association before 2009. Second, and related, is the short time interval between waves in our study. Year-to-year neighborhood changes likely presented fewer significant influences of gentrification on crime compared with a large time interval (e.g., a ten-year interval), but the short interval enabled us to have a more conservative estimate of the effect and capture the nuances in this association during the transitional time. Further, a shift from not gentrified to gentrified from one year to the next may seem inconsistent with the conceptualization of gentrification as a gradual process. Although we attended to this in multiple ways in sensitivity models available from the authors (e.g., using 2-year windows instead of 1-year windows and ensuring the point of gentrification signaled real changes in trajectories of neighborhood characteristics rather than a temporary deviation), these annual data likely mask some neighborhood change. At the same time, our findings are consistent with studies examining the relationship between gentrification and other, presumably less responsive, life outcomes. For instance, Gibbons, Bartons, and Brault [47] identified the protective effect of gentrification on self-rated health at the neighborhood level. The stability of these effects across both shorter term (crime) and longer term (health) outcomes enhanced the validity of the measure of gentrification.

Third, our approach employed within-tract effects consistent with previous studies [21,24] to better rule out spuriousness (i.e., that both gentrification and crime are attributable to some unmeasured difference between neighborhoods), yet other approaches that can more rigorously assess causality are needed in future research. These approaches might entail capitalizing on natural experiments via policy change or employing propensity matching approach that first entails predicting the likelihood of gentrification and then matching tracts on this likelihood. These approaches were beyond the scope and data capabilities of the current study.

Fourth, there are drawbacks to using census tracts to define neighborhoods. Although we tested for spatial lag and spatial error only to find nonsignificant spatial dependence (both lag and error) for property and violent crime, these and other spatial effects are important for future consideration. For example, the crime rates in gentrifying tracts where nearby neighborhoods are also improving may differ from the crime rates in those on the “frontier” of gentrification [22], or those standing as an “island of renewal in a sea of decay” [61]. Also, the spatial effect of gentrified neighborhoods and disadvantaged neighborhoods can spill over into adjacent areas. Using census tracts to study neighborhoods, even while attending to potential spatial lag and error, does not entirely consider the blurred boundaries between neighborhoods. Individuals may live in one census tract while spending most of their daytime in another tract for work or entertainment. However, using a crude indicator of neighborhoods like census tracts likely provided a more conservative estimate of the link between gentrification and crime.

Finally, our measure of crime rate is limited in at least two ways. First, as is the case with any study using official police reports of crime, our dependent variable conflates both actual occurrences of crime and the reporting of that crime. Second, crime rates only take into account actual residents versus those who visit the neighborhood for restaurants, bars, shops, or other venues. As visitors are more frequent in gentrifying neighborhoods versus vulnerable neighborhoods [62], crime rates in gentrified neighborhoods might actually be overestimated in the current study. Nonetheless, we found consistent negative associations between gentrification and crime over time despite evidence that middle-class in-movers demand more frequent policing in gentrifying communities [39,40,60] and despite increased foot traffic in gentrified neighborhoods. Hence, the findings presented here may be conservative estimates rather than liberal estimates of the association between gentrification and crime.

In light of these limitations, we found gentrification was consistently related to reduced property crime in Buffalo from 2011 to 2019 across three different model specifications and had no effect on violent crime. However, it is important to consider this evidence as only one piece of the general puzzle about the effects of gentrification [6]. Understanding the real-world implications of these findings requires both replication in other mid-sized cities and the placing of these findings in context. It is important to consider that gentrification is a multidimensional process that has also been related to negative neighborhood consequences, like the displacement of disadvantaged residents, especially among people of color [14]. It is also important to keep in mind that crime rates were declining in Buffalo prior to widespread gentrification. Although some of this decline could be capturing the gentrification process, others have identified non-gentrification-related factors responsible for general declines in crime across the U.S. [63,64] Hence, urban planners must consider the entirety of research on gentrification and its consequences for diverse outcomes and diverse groups. Doing so may point to novel ways to capitalize on the crime-reducing benefits of gentrification, as well as emerging evidence on its general health-promoting benefits [47,65], while minimizing costs to marginalized populations.

## Supporting information

S1 Fig Property crime trajectories among tracts by gentrification status using city-level comparisons. (TIF)

S2 Fig Property crime rate by time from gentrification among gentrified tracts using city-level comparisons. (TIF)

S1 Table Descriptive statistics for neighborhood variables at the tract level from 2011 to 2019 (compared with city level). (DOCX)

## References

[1] Bhavsar NA, Kumar M, Richman L. Defining gentrification for epidemiologic research: A systematic review. PLOS ONE. 2020 May 21;15(5):e0233361. doi: 10.1371/journal.pone.0233361 32437388 PMC7241805

[2] Freeman L. Displacement or Succession?: Residential Mobility in Gentrifying Neighborhoods. Urban Aff Rev. 2005 Mar 1;40(4):463–91.

[3] Kirkland E. What’s Race Got to Do With it? Looking for the Racial Dimensions of Gentrifícation. West J Black Stud. 2008;32(2):14.

[4] Phillips D, Flores L, Henderson J. Development without Displacement: Resisting Gentrification in the Bay Area [Internet]. Northern California Grantmakers. 2014 [cited 2021 Nov 9]. Available from: https://ncg.org/resources/development-without-displacement-resisting-gentrification-bay-area.

[5] Smith N. Toward a Theory of Gentrification A Back to the City Movement by Capital, not People. J Am Plann Assoc. 1979 Oct 1;45(4):538–48.

[6] Brown-Saracino J. Explicating Divided Approaches to Gentrification and Growing Income Inequality. Annu Rev Sociol. 2017;43(1):515–39.

[7] Taylor MM. Can You Go Home Again? Black Gentrification and the Dilemma of Difference. Berkeley J Sociol. 1992;37:101–28.

[8] Hwang J. Pioneers of Gentrification: Transformation in Global Neighborhoods in Urban America in the Late Twentieth Century. Demography. 2016;53(1):189–213. doi: 10.1007/s13524-015-0448-4 26689938 PMC4742432

[9] Kennedy M, Leonard P. Dealing with neighborhood change: A primer on gentrification and policy choices. Brookings Institution Center on Urban and Metropolitan Policy Washington, DC; 2001.

[10] Zukin S. The cultures of cities Blackwell. Camb MA. 1995.

[11] Zukin S, Trujillo V, Frase P, Jackson D, Recuber T, Walker A. New Retail Capital and Neighborhood Change: Boutiques and Gentrification in New York City. City Community. 2009 Mar 1;8(1):47–64.

[12] Bates L. Gentrification and Displacement Study: Implementing an Equitable Inclusive Development Strategy in the Context of Gentrification. Urban Stud Plan Fac Publ Present [Internet]. 2013 May 1; Available from: https://pdxscholar.library.pdx.edu/usp_fac/83.

[13] Hartman C. Comment on “Neighborhood Revitalization and Displacement: A Review of the Evidence.” J Am Plann Assoc. 1979 Oct;45(4):488–91.

[14] Hwang J, Ding L. Unequal Displacement: Gentrification, Racial Stratification, and Residential Destinations in Philadelphia. Am J Sociol. 2020 Sep 1;126(2):354–406.

[15] Barton M. Gentrification and Violent Crime in New York City. Crime Delinquency. 2016;62(9):1180–202.

[16] Covington J, Taylor RB. Gentrification and Crime: Robbery and Larceny Changes in Appreciating Baltimore Neighborhoods during the 1970s. Urban Aff Q. 1989 Sep 1;25(1):142–72.

[17] Lee YY. Gentrification and Crime: Identification Using the 1994 Northridge Earthquake in Los Angeles. J Urban Aff. 2010;32(5):549–77.

[18] MacDonald JM, Stokes RJ. Gentrification, Land Use, and Crime. Annu Rev Criminol. 2020;3(1):121–38.

[19] Papachristos AV, Smith CM, Scherer ML, Fugiero MA. More Coffee, Less Crime? The Relationship between Gentrification and Neighborhood Crime Rates in Chicago, 1991 to 2005. City Community. 2011;10(3):215–40.

[20] Barton M. An exploration of the importance of the strategy used to identify gentrification. Urban Stud. 2016 Jan 1;53(1):92–111.

[21] Barton MS, Valasik MA, Brault E, Tita G. “Gentefication” in the Barrio: Examining the Relationship Between Gentrification and Homicide in East Los Angeles. Crime Delinquency. 2020 Dec 1;66(13–14):1888–913.

[22] Boggess LN, Hipp JR. The Spatial Dimensions of Gentrification and the Consequences for Neighborhood Crime. Justice Q. 2016 Jun 6;33(4):584–613.

[23] Ocejo RE, Kosta EB, Mann A. Centering small cities for urban sociology in the 21st century. Vol. 19, City & Community. SAGE Publications Sage CA: Los Angeles, CA; 2020.

[24] Kreager DA, Lyons CJ, Hays ZR. Urban Revitalization and Seattle Crime, 1982−2000. Soc Probl. 2011 Nov;58(4):615–39. doi: 10.1525/sp.2011.58.4.615 25505350 PMC4259159

[25] McDonald SC. Does Gentrification Affect Crime Rates? Crime Justice. 1986 Jan 1;8:163–201.

[26] Coley J, Adelman RM. Gentrification in the “City of Good Neighbors”: Race, Class, and Neighborhoods in Buffalo. Sociol Inq. 2021;91(4):824–48.

[27] Silverman RM, Taylor HL Jr, Yin L, Miller C, Buggs P. Place making as a form of place taking: Residential displacement and grassroots resistance to institutional encroachment in Buffalo, New York. J Place Manag Dev. 2019.

[28] Shaw CR, McKay HD. Juvenile delinquency and urban areas. Chicago, IL, US: University of Chicago Press; 1942. xxxii, 451 p. (Juvenile delinquency and urban areas).

[29] Atkinson R. The evidence on the impact of gentrification: new lessons for the urban renaissance? Eur J Hous Policy. 2004 Jan 1;4(1):107–31.

[30] Morenoff JD, Sampson RJ. Violent Crime and The Spatial Dynamics of Neighborhood Transition: Chicago, 1970–1990. Soc Forces. 1997;76(1):31–64.

[31] Wyly EK, Hammel DJ. Islands of Decay in Seas of Renewal: Housing Policy and the Resurgence of Gentrification. 1999.

[32] Blau P. Inequality and heterogeneity: A primitive theory of social structure. Vol. 7. Free Press New York; 1977.

[33] Kirk DS, Laub JH. Neighborhood Change and Crime in the Modern Metropolis. Crime Justice. 2010 Jan 1;39:441–502.

[34] Legewie J, Schaeffer M. Contested Boundaries: Explaining Where Ethnoracial Diversity Provokes Neighborhood Conflict. Am J Sociol. 2016 Jul 1;122(1):125–61. doi: 10.1086/686942 29873459

[35] Armstrong TA, Katz CM, Schnebly SM. The Relationship Between Citizen Perceptions of Collective Efficacy and Neighborhood Violent Crime. Crime Delinquency. 2015;61(1):121–42.

[36] Sampson RJ. Great American City: Chicago and the Enduring Neighborhood Effect [Internet]. Great American City. University of Chicago Press; 2021 [cited 2021 Oct 29]. Available from: https://www.degruyter.com/document/doi/10.7208/9780226733883/html

[37] Sampson RJ, Raudenbush SW, Earls F. Neighborhoods and Violent Crime: A Multilevel Study of Collective Efficacy. Science. 1997 Aug 15;277(5328):918–24. doi: 10.1126/science.277.5328.918 9252316

[38] Cohen LE, Felson M. Social Change and Crime Rate Trends: A Routine Activity Approach. Am Sociol Rev. 1979;44(4):588–608.

[39] Freeman L. There goes the hood: Views of gentrification from the ground up. Temple University Press; 2006.

[40] Parekh T. “They want to live in the Tremé, but they want it for their ways of living”: gentrification and neighborhood practice in Tremé, New Orleans. Urban Geogr. 2015 Feb 17;36(2):201–20.

[41] Patch J. “Ladies and gentrification”: New stores, residents, and relationships in neighborhood change. Emerald Group Publishing Limited; 2008.

[42] Aliprantis D, Hartley D. Blowing it up and knocking it down: The local and city-wide effects of demolishing high concentration public housing on crime. J Urban Econ. 2015 Jul;88:67–81.

[43] Smith CM. The influence of gentrification on gang homicides in Chicago neighborhoods, 1994 to 2005. Crime Delinquency. 2014;60(4):569–91.

[44] Hackworth J. Public housing and the rescaling of regulation in the USA. Environ Plan A. 2003;35(3):531–49.

[45] Kawachi I, Kennedy BP, Wilkinson RG. Crime: social disorganization and relative deprivation. Soc Sci Med. 1999;48(6):719–31. doi: 10.1016/s0277-9536(98)00400-6 10190635

[46] Barnett C, Mencken FC. Social Disorganization Theory and the Contextual Nature of Crime in Nonmetropolitan Counties*. Rural Sociol. 2002;67(3):372–93.

[47] Gibbons J, Barton M, Brault E. Evaluating gentrification’s relation to neighborhood and city health. PLOS ONE. 2018 Nov 19;13(11):e0207432. doi: 10.1371/journal.pone.0207432 30452460 PMC6242354

[48] Hobor G. Surviving the era of deindustrialization: The new economic geography of the urban rust belt. J Urban Aff. 2013;35(4):417–34.

[49] Pitegoff PR. Buffalo Change & Community. Buff Rev. 1991;39:313.

[50] Perry DC, McLean B. Aftermath of Deindustrialization: The Meaning of Economic Restructuring in Buffalo, New York, The. Buff Rev. 1991;39:345.

[51] Koritz D. Restructuring or destructuring? Deindustrialization in two industrial heartland cities. Urban Aff Q. 1991;26(4):497–511.

[52] Adelman RM, Balta Ozgen A, Rabii W. Buffalo’s West Side Story: Migration, Gentrification, and Neighborhood Change. City Community. 2019 Sep 1;18(3):770–91.

[53] Silverman RM, Lewis J, Patterson KL. William Worthy’s Concept of “Institutional Rape” Revisited: Anchor Institutions and Residential Displacement in Buffalo, NY. Humanity Soc. 2014 May;38(2):158–81.

[54] Frey WH. Where Immigrant Growth Matters Most. Brookings. 2017.

[55] Swanstrom T, Plöger J. What to Make of Gentrification in Older Industrial Cities? Comparing St. Louis (USA) and Dortmund (Germany). Urban Aff Rev. 2022 Mar 1;58(2):526–62.

[56] Morgan RE, Thompson A. The nation’s two crime measures, 2011–2020. Bur Justice Stat Httpsbjs Ojp Govcontentpubpdfntcm1120 Pdf Accessed April 2022. 2022.

[57] Walker K, Herman M. tidycensus. 2024. Available from: Available: https://cran.r-project.org/web/packages/tidycensus/tidycensus.pdf.

[58] Osgood DW. Poisson-based regression analysis of aggregate crime rates. J Quant Criminol. 2000;16:21–43.

[59] Laniyonu A. Coffee Shops and Street Stops: Policing Practices in Gentrifying Neighborhoods. Urban Aff Rev. 2018 Sep 1;54(5):898–930.

[60] Beck B. Policing Gentrification: Stops and Low–Level Arrests during Demographic Change and Real Estate Reinvestment. City Community. 2020 Mar 1;19(1):245–72.

[61] Berry BJ. Islands of renewal in seas of decay. New Urban Real. 1985;69–96.

[62] Burnett K. Commodifying poverty: gentrification and consumption in Vancouver’s Downtown Eastside. Urban Geogr. 2014 Feb 17;35(2):157–76.

[63] Farrell G, Tilley N, Tseloni A. Why the Crime Drop? Crime Justice. 2014 Sep;43:421–90.

[64] Levitt SD. Understanding Why Crime Fell in the 1990s: Four Factors that Explain the Decline and Six that Do Not. J Econ Perspect. 2004 Mar;18(1):163–90.

[65] Gibbons J, Barton MS. The Association of Minority Self-Rated Health with Black versus White Gentrification. J Urban Health. 2016 Dec 1;93(6):909–22. doi: 10.1007/s11524-016-0087-0 27761683 PMC5126023

