# Supplementary figures and images for: Gentrification and crime in Buffalo, New York
Source: PLoS One. 2024 Jun 20;19(6):e0302832. doi: 10.1371/journal.pone.0302832 (PMC11189242; doi:10.1371/journal.pone.0302832)

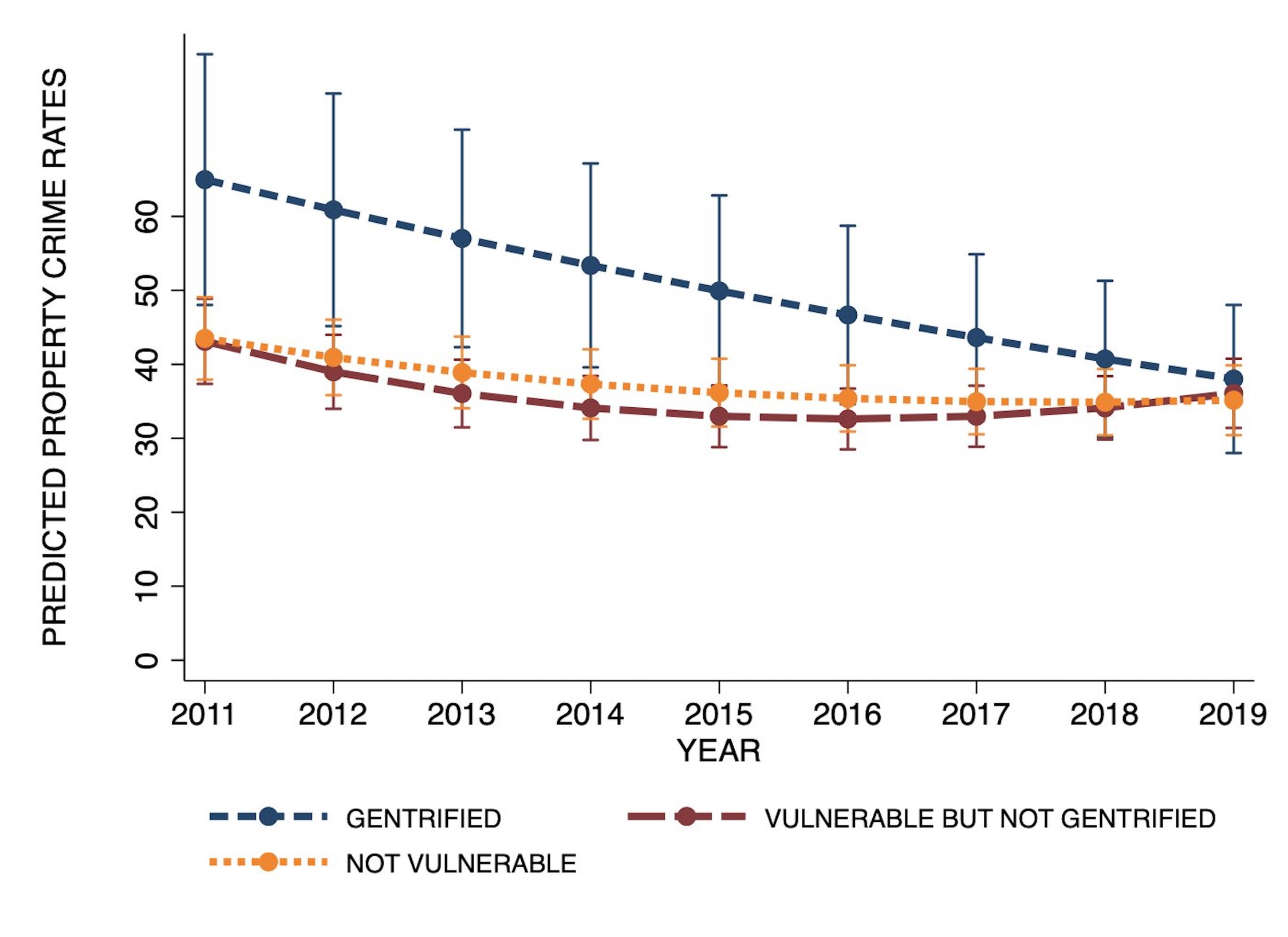

Supplement: S1 Fig — (TIF) [file pone.0302832.s001.tif]

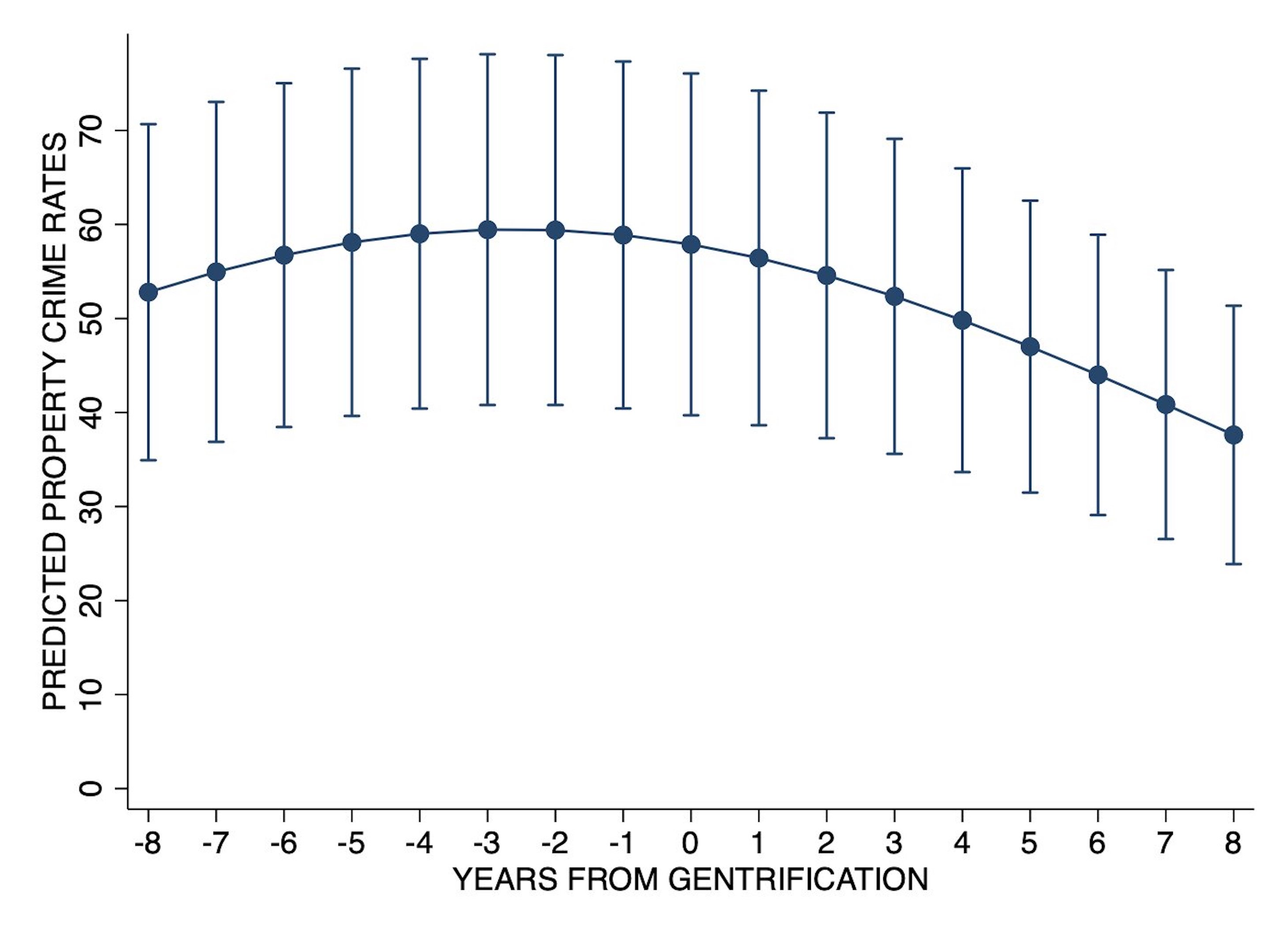

Supplement: S2 Fig — (TIF) [file pone.0302832.s002.tif]
