# Supplementary material for: Gentrification and crime in Buffalo, New York
Source: PLoS One. 2024 Jun 20;19(6):e0302832. doi: 10.1371/journal.pone.0302832 (PMC11189242; doi:10.1371/journal.pone.0302832)
Supplement: S1 Table — (DOCX) [file pone.0302832.s003.docx]

**S1 Table.** **Descriptive Statistics for Neighborhood Variables at the Tract Level from 2011 to 2019 (Compared with City Level)**

|  |  | **Gentrified tracts** | | | | **Vulnerable tracts** | | | | **Advantaged tracts** | | | |
| --- | --- | --- | --- | --- | --- | --- | --- | --- | --- | --- | --- | --- | --- |
| **Year** | **Gentrified counts (%)** | **Concentrated disadvantage** | **Residential stability** | **% Foreign-born** | **% Youth** | **Concentrated disadvantage** | **Residential stability** | **% Foreign-born** | **% Youth** | **Concentrated disadvantage** | **Residential stability** | **% Foreign-born** | **% Youth** |
| 2011 | 1 (1.27) | -0.13 | -0.29 | 2.67 | 27.44 | 0.83 | -0.33 | 3.42 | 16.18 | -0.50 | 0.35 | 2.07 | 16.87 |
| 2012 | 2 (2.53) | -0.06 | 0.07 | 3.93 | 22.07 | 0.91 | -0.41 | 4.01 | 17.07 | -0.42 | 0.34 | 2.24 | 16.84 |
| 2013 | 3 (3.80) | 0.45 | -0.44 | 10.34 | 21.18 | 0.82 | -0.49 | 3.72 | 17.13 | -0.35 | 0.33 | 2.28 | 16.15 |
| 2014 | 4 (5.06) | 0.32 | -0.37 | 6.84 | 23.40 | 0.65 | -0.50 | 3.88 | 16.44 | -0.43 | 0.32 | 2.59 | 16.49 |
| 2015 | 7 (8.86) | 0.29 | -0.25 | 5.99 | 17.50 | 0.59 | -0.39 | 4.34 | 16.87 | -0.52 | 0.32 | 2.35 | 15.51 |
| 2016 | 7 (8.86) | 0.24 | -0.29 | 6.73 | 15.66 | 0.52 | -0.43 | 5.02 | 16.40 | -0.48 | 0.31 | 2.08 | 15.62 |
| 2017 | 10 (12.66) | 0.12 | -0.40 | 7.06 | 15.72 | 0.50 | -0.35 | 4.60 | 16.01 | -0.65 | 0.30 | 2.21 | 14.87 |
| 2018 | 13 (16.46) | 0.10 | -0.46 | 8.13 | 16.17 | 0.49 | -0.27 | 3.31 | 15.25 | -0.71 | 0.25 | 2.60 | 14.41 |
| 2019 | 14 (17.72) | 0.06 | -0.27 | 8.09 | 15.41 | 0.51 | -0.31 | 3.86 | 14.90 | -0.72 | 0.34 | 2.52 | 14.34 |

**S2 Table. Random Intercept Models Predicting Property Crime Rates Among All Tracts**

|  | Model 1 | Model 2 | Model 3 |
| --- | --- | --- | --- |
| **Tract-level Predictors** | |  |  |
| Gentrification stage (ref = vulnerable but not gentrified) |  |  |  |
| Gentrified | 0.334* | 0.352* | 0.353* |
|  | (0.147) | (0.156) | (0.148) |
| Not vulnerable | -0.002 | -0.043 | -0.002 |
|  | (0.047) | (0.067) | (0.049) |
| Concentrated disadvantage | 0.133 | 0.157 | 0.155 |
|  | (0.088) | (0.088) | (0.091) |
| Residential stability | -0.225* | -0.231* | -0.211 |
|  | (0.106) | (0.107) | (0.108) |
| % Foreign born | -0.021 | -0.021 | -0.011 |
|  | (0.014) | (0.014) | (0.015) |
| % Youth | -0.006 | -0.006 | -0.024** |
|  | (0.008) | (0.008) | (0.008) |
| Year | -0.096*** | -0.134*** | -0.096*** |
|  | (0.010) | (0.017) | (0.010) |
| Year^2^ | 0.006*** | 0.011*** | 0.007*** |
|  | (0.001) | (0.002) | (0.001) |
| Gentrified x Year | | 0.070** |  |
|  |  | (0.026) |  |
| Not vulnerable x Year |  | 0.059* |  |
|  |  | (0.024) |  |
| Gentrified x Year^2^ | | -0.011*** |  |
|  |  | (0.003) |  |
| Not vulnerable x Year^2^ |  | -0.006* |  |
|  |  | (0.002) |  |
| **Time-varying predictors** | |  |  |
| Gentrified in a given year | |  | -0.048 |
|  |  |  | (0.035) |
| Concentrated disadvantage | | | -0.022 |
|  |  |  | (0.026) |
| Residential stability | |  | -0.016 |
|  |  |  | (0.019) |
| % Foreign born | |  | -0.010* |
|  |  |  | (0.005) |
| % Youth |  |  | 0.017*** |
|  |  |  | (0.002) |
| constant | 3.963*** | 3.961*** | 3.942*** |
|  | (0.147) | (0.150) | (0.147) |
|  | | |  |
| * p<0.05 ** p<0.01 *** p<0.001. N = 693 tract-years across 77 tracts  Notes. Standard errors in parentheses. | | | |

**S1 Fig. Property Crime Trajectories among Tracts by Gentrification Status Using City-level Comparisons**

**S3 Table. Random Intercept Model Predicting Property Crime Rates Among Gentrified Tracts**

|  | Property Crime Rate |
| --- | --- |
| Years from gentrification | -0.021* |
|  | (0.008) |
| Years from gentrification^2^ | -0.004*** |
|  | (0.001) |
| Year gentrified | -0.150* |
|  | (0.063) |
| Concentrated disadvantage | 0.195*** |
|  | (0.052) |
| Residential stability | 0.085 |
|  | (0.044) |
| % Foreign-born | -0.021** |
|  | (0.008) |
| % Youth | 0.015*** |
|  | (0.004) |
| Constant | 306.226 |
|  | (126.382) |
| N = 126 tract-years across 14 gentrified tracts | |
| Standard errors in parentheses | |
| * p<0.05 ** p<0.01 *** p<0.001 | |

**S2 Fig: Property Crime Rate by Time from Gentrification among Gentrified Tracts Using City-level Comparisons**
